# 2,4,6,8-Tetra­kis(4-ethyl­phen­yl)-3,7-diaza­bicyclo­[3.3.1]nonan-9-one

**DOI:** 10.1107/S1600536810016569

**Published:** 2010-05-12

**Authors:** K. Rajesh, V. Vijayakumar, A. P. Safwan, Kong Wai Tan, Edward R. T. Tiekink

**Affiliations:** a Organic Chemistry Division, School of Advanced Sciences, VIT University, Vellore 632 014, India; b Department of Chemistry, University of Malaya, 50603 Kuala Lumpur, Malaysia

## Abstract

The bicyclo­[3.3.1]nonane ring in the title compound, C_39_H_44_N_2_O, adopts a chair–boat conformation with the four benzene rings being directed away from the carbonyl group. The presence of C—H⋯O contacts leads to helical supra­molecular chains along the *b* axis.

## Related literature

For background to the synthesis and stereochemistry of 3,7-diaza­bicyclo­[3.3.1]nonan-9-ones and their derivatives, see: Srikrishna & Vijaykumar (1998 ▶); Pathak *et al.* (2007 ▶); Vijayakumar & Sundaravadivelu (2005 ▶). For related structures, see: Natarajan *et al.* (2008 ▶); Fun *et al.* (2009 ▶). For conformational analysis, see: Cremer & Pople (1975 ▶).
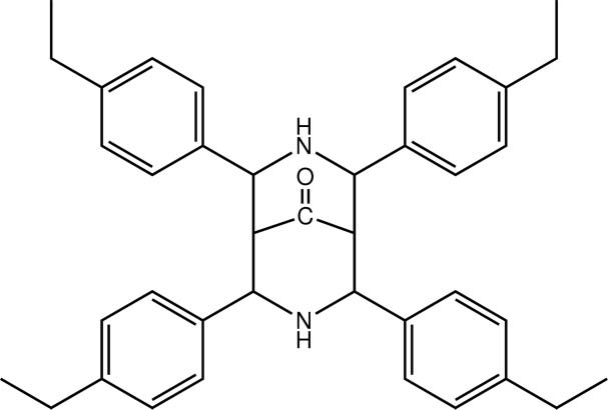


## Experimental

### 

#### Crystal data

C_39_H_44_N_2_O
*M* *_r_* = 556.76
Monoclinic, 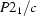

*a* = 13.381 (2) Å
*b* = 11.8217 (17) Å
*c* = 19.989 (3) Å
β = 99.675 (4)°
*V* = 3117.1 (8) Å^3^
*Z* = 4
Mo *K*α radiation
μ = 0.07 mm^−1^
*T* = 100 K
0.40 × 0.37 × 0.29 mm

#### Data collection

Bruker SMART APEX diffractometer
Absorption correction: multi-scan (*SADABS*; Sheldrick, 1996 ▶) *T*_min_ = 0.972, *T*_max_ = 0.980
29235 measured reflections
7159 independent reflections
5783 reflections with *I* > 2σ(*I*)
*R*_int_ = 0.033

#### Refinement

*R*[*F* ^2^ > 2σ(*F* ^2^)] = 0.042
*wR*(*F* ^2^) = 0.117
*S* = 1.03
7159 reflections
389 parameters
2 restraints
H atoms treated by a mixture of independent and constrained refinement
Δρ_max_ = 0.33 e Å^−3^
Δρ_min_ = −0.27 e Å^−3^

### 

Data collection: *APEX2* (Bruker, 2008 ▶); cell refinement: *SAINT* (Bruker, 2008 ▶); data reduction: *SAINT*; program(s) used to solve structure: *SHELXS97* (Sheldrick, 2008 ▶); program(s) used to refine structure: *SHELXL97* (Sheldrick, 2008 ▶); molecular graphics: *ORTEP-3* (Farrugia, 1997 ▶) and *DIAMOND* (Brandenburg, 2006 ▶); software used to prepare material for publication: *publCIF* (Westrip, 2010 ▶).

## Supplementary Material

Crystal structure: contains datablocks global, I. DOI: 10.1107/S1600536810016569/hg2681sup1.cif

Structure factors: contains datablocks I. DOI: 10.1107/S1600536810016569/hg2681Isup2.hkl

Additional supplementary materials:  crystallographic information; 3D view; checkCIF report

## Figures and Tables

**Table 1:** Hydrogen-bond geometry (Å, °)

*D*—H⋯*A*	*D*—H	H⋯*A*	*D*⋯*A*	*D*—H⋯*A*
C26—H26⋯O1^i^	0.95	2.51	3.3837 (16)	153

Symmetry code: (i) 
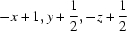
.

**Fig. 1.**
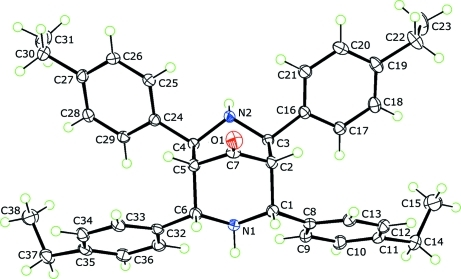
The molecular structure of (I) showing the atom-labelling scheme and displacement ellipsoids at the 50% probability level.

**Fig. 2.**
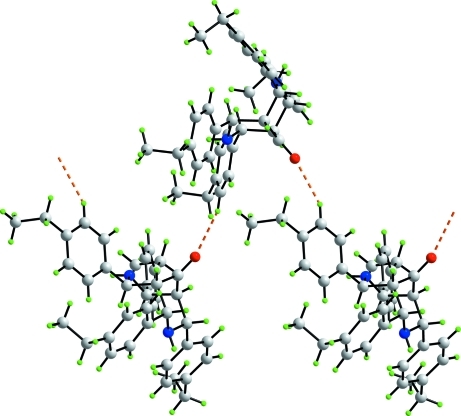
Helical supramolecular chain along the b axis in (I) mediated by C–H···O contacts, shown as orange dashed lines.

## supplementary crystallographic information

### Comment

The synthesis and stereochemistry of 3,7-diazabicyclo[3.3.1]nonan-9-ones and their derivatives are of much interest owing to their diverse biological activities (Srikrishna & Vijaykumar, 1998; Pathak *et al.*, 2007). The conformational analysis of 3,7-diazabicyclo[3.3.1]nonanes (bispidines) is of interest from a theoretical view point and in particular the 2,4,6,8-tetraaryl-3,7-diazabicyclo[3.3.1]nonanes constitute an interesting case for study owing to the presence of four aryl groups (Vijayakumar & Sundaravadivelu, 2005). If all aryl groups occupy equatorial orientations, molecular models indicate their close proximity to both rings in the bicyclic systems. By contrast, if they are in the twin chair conformation, severe non-bonded interactions arise between aryl groups occupying 2,8-positions and 4,6-positions. In the present report, in continuation of studies in this area (Natarajan *et al.*, 2008; Fun *et al.*, 2009), the synthesis and structure determination of a new example, the title compound (I), is described.

In (I), the bicyclo[3.3.1]nonane ring adopts a chair-boat conformation with ring puckering amplitudes (Cremer & Pople, 1975) for the N1-containing ring (chair) being Q = 0.6318 (13) Å, θ = 6.38 (11) ° and φ = 183.5 (11) °. For the N2-ring, which adopts the boat form, the equivalent parameters are 0.8044 (12) Å, 88.29 (9) °, and 358.47 (9) °, respectively. The benzene rings adjacent to the N1 atom are each directed away from the carbonyl group and are effectively co-planar [dihedral angle = 6.91 (6) °]. The arrangement defines a planar facade to this side of the molecule, especially considering the ethyl groups are folded back to be orientated toward the rest of the molecule. By contrast, the benzene rings adjacent to the N2 atom are somewhat splayed with adjacent benzene rings forming dihedral angles of 54.17 (6) ° [(C8–C13)/(C16–C21)] and 48.45 (6) ° [(C24–C29)/(C32–C37)]. The dihedral angle between the (C16–C21) and (C24–C29) rings is 38.01 (6) ° so as to define a concave facade to this part of the molecule; the ethyl groups for these benzene rings are directed away from the molecule.

Despite there being two acidic N—H H atoms in the structure, neither play a significant role in the crystal packing owing to steric congestion. Rather, the carbonyl group participates in a C–H···O contact, Table 1, to generate a supramolecular chain with helical topology along the *b* axis, Fig. 2.

### Experimental

A mixture of acetone (0.2 ml), 4-ethylbenzaldehyde (2 ml) and dry ammonium acetate (0.6 g) were taken in a 1:4:2 molar ratio in ethanol (15 ml) and the resulting solution heated on water bath till the colour changed to red-orange. The mixture was allowed to stand for 24 h. The resultant sticky precipitate was washed with a mixture of diethyl ether and ethanol (4:1). The solid obtained was crystallized from a mixture of CHCl_3_-methanol (1:1) to yield (I). M.Pt: 495–497 K.

### Refinement

Carbon-bound H-atoms were placed in calculated positions (C—H 0.95 to 0.99 Å) and were included in the refinement in the riding model approximation, with *U*_iso_(H) set to 1.2 to 1.5*U*_equiv_(C). The amine-H atoms were refined with the distance restraint N–H = 0.91±0.1 Å, and with *U*_iso_(H) = 1.2*U*_equiv_(N).

### Crystal data

**Table:**

C_39_H_44_N_2_O	*F*(000) = 1200
*M**_r_* = 556.76	*D*_x_ = 1.186 Mg m^−^^3^
Monoclinic, *P*2_1_/*c*	Mo *K*α radiation, λ = 0.71073 Å
Hall symbol: -P 2ybc	Cell parameters from 9317 reflections
*a* = 13.381 (2) Å	θ = 2.3–28.2°
*b* = 11.8217 (17) Å	µ = 0.07 mm^−^^1^
*c* = 19.989 (3) Å	*T* = 100 K
β = 99.675 (4)°	Block, colourless
*V* = 3117.1 (8) Å^3^	0.40 × 0.37 × 0.29 mm
*Z* = 4	

### Data collection

**Table:**

Bruker SMART APEX diffractometer	7159 independent reflections
Radiation source: fine-focus sealed tube	5783 reflections with *I* > 2σ(*I*)
graphite	*R*_int_ = 0.033
ω scans	θ_max_ = 27.5°, θ_min_ = 1.5°
Absorption correction: multi-scan (*SADABS*; Sheldrick, 1996)	*h* = −17→17
*T*_min_ = 0.972, *T*_max_ = 0.980	*k* = −15→15
29235 measured reflections	*l* = −25→25

### Refinement

**Table:**

Refinement on *F*^2^	Primary atom site location: structure-invariant direct methods
Least-squares matrix: full	Secondary atom site location: difference Fourier map
*R*[*F*^2^ > 2σ(*F*^2^)] = 0.042	Hydrogen site location: inferred from neighbouring sites
*wR*(*F*^2^) = 0.117	H atoms treated by a mixture of independent and constrained refinement
*S* = 1.03	*w* = 1/[σ^2^(*F*_o_^2^) + (0.0602*P*)^2^ + 0.9409*P*] where *P* = (*F*_o_^2^ + 2*F*_c_^2^)/3
7159 reflections	(Δ/σ)_max_ < 0.001
389 parameters	Δρ_max_ = 0.33 e Å^−^^3^
2 restraints	Δρ_min_ = −0.27 e Å^−^^3^

### Special details

**Table:**

Geometry. All s.u.'s (except the s.u. in the dihedral angle between two l.s. planes) are estimated using the full covariance matrix. The cell s.u.'s are taken into account individually in the estimation of s.u.'s in distances, angles and torsion angles; correlations between s.u.'s in cell parameters are only used when they are defined by crystal symmetry. An approximate (isotropic) treatment of cell s.u.'s is used for estimating s.u.'s involving l.s. planes.
Refinement. Refinement of *F*^2^ against ALL reflections. The weighted *R*-factor wR and goodness of fit *S* are based on *F*^2^, conventional *R*-factors *R* are based on *F*, with *F* set to zero for negative *F*^2^. The threshold expression of *F*^2^ > 2σ(*F*^2^) is used only for calculating *R*-factors(gt) etc. and is not relevant to the choice of reflections for refinement. *R*-factors based on *F*^2^ are statistically about twice as large as those based on *F*, and *R*- factors based on ALL data will be even larger.

### Fractional atomic coordinates and isotropic or equivalent isotropic displacement parameters (Å^2^)

**Table:**

	*x*	*y*	*z*	*U*_iso_*/*U*_eq_	
O1	0.28740 (7)	0.33935 (8)	0.24139 (5)	0.0258 (2)	
N1	0.10571 (8)	0.43495 (9)	0.35954 (5)	0.0176 (2)	
H1N	0.0682 (12)	0.4050 (13)	0.3883 (8)	0.026*	
N2	0.22518 (7)	0.62144 (9)	0.25571 (5)	0.0159 (2)	
H2N	0.2198 (11)	0.6951 (14)	0.2501 (7)	0.024*	
C1	0.06877 (9)	0.39020 (10)	0.29151 (6)	0.0171 (2)	
H1	0.0782	0.3063	0.2923	0.020*	
C2	0.13203 (9)	0.44252 (10)	0.24037 (6)	0.0158 (2)	
H2	0.1105	0.4076	0.1946	0.019*	
C3	0.12289 (9)	0.57417 (10)	0.23417 (6)	0.0149 (2)	
H3	0.0769	0.6024	0.2651	0.018*	
C4	0.26318 (9)	0.59657 (10)	0.32788 (6)	0.0149 (2)	
H4	0.2114	0.6212	0.3556	0.018*	
C5	0.27729 (9)	0.46582 (10)	0.33456 (6)	0.0160 (2)	
H5	0.3506	0.4477	0.3498	0.019*	
C6	0.21310 (9)	0.41261 (10)	0.38478 (6)	0.0164 (2)	
H6	0.2243	0.3290	0.3863	0.020*	
C7	0.24032 (9)	0.41035 (10)	0.26704 (6)	0.0172 (2)	
C8	−0.04266 (9)	0.41626 (10)	0.26868 (6)	0.0175 (2)	
C9	−0.09358 (9)	0.49827 (11)	0.29977 (6)	0.0213 (3)	
H9	−0.0603	0.5344	0.3398	0.026*	
C10	−0.19329 (9)	0.52824 (12)	0.27270 (7)	0.0234 (3)	
H10	−0.2269	0.5843	0.2947	0.028*	
C11	−0.24389 (9)	0.47706 (11)	0.21398 (6)	0.0214 (3)	
C12	−0.19308 (10)	0.39341 (11)	0.18375 (6)	0.0222 (3)	
H12	−0.2265	0.3567	0.1439	0.027*	
C13	−0.09445 (10)	0.36279 (10)	0.21087 (6)	0.0204 (3)	
H13	−0.0618	0.3047	0.1898	0.025*	
C14	−0.34976 (10)	0.51291 (12)	0.18224 (7)	0.0267 (3)	
H14A	−0.3800	0.5581	0.2154	0.032*	
H14B	−0.3923	0.4448	0.1709	0.032*	
C15	−0.34962 (12)	0.58247 (14)	0.11839 (9)	0.0382 (4)	
H15A	−0.3103	0.6518	0.1298	0.057*	
H15B	−0.4194	0.6022	0.0985	0.057*	
H15C	−0.3190	0.5383	0.0856	0.057*	
C16	0.08053 (9)	0.60808 (10)	0.16182 (6)	0.0162 (2)	
C17	−0.02427 (9)	0.61354 (10)	0.14070 (6)	0.0183 (2)	
H17	−0.0682	0.6043	0.1730	0.022*	
C18	−0.06514 (10)	0.63238 (11)	0.07294 (6)	0.0216 (3)	
H18	−0.1366	0.6361	0.0597	0.026*	
C19	−0.00307 (10)	0.64589 (10)	0.02430 (6)	0.0221 (3)	
C20	0.10134 (10)	0.64380 (11)	0.04593 (6)	0.0230 (3)	
H20	0.1452	0.6552	0.0138	0.028*	
C21	0.14298 (10)	0.62529 (11)	0.11384 (6)	0.0205 (3)	
H21	0.2145	0.6245	0.1273	0.025*	
C22	−0.04871 (12)	0.66010 (12)	−0.04984 (7)	0.0295 (3)	
H22A	0.0023	0.6378	−0.0780	0.035*	
H22B	−0.1072	0.6082	−0.0609	0.035*	
C23	−0.08338 (14)	0.77868 (13)	−0.06826 (8)	0.0440 (4)	
H23A	−0.1328	0.8022	−0.0400	0.066*	
H23B	−0.1149	0.7813	−0.1162	0.066*	
H23C	−0.0250	0.8299	−0.0607	0.066*	
C24	0.36114 (9)	0.66011 (10)	0.35158 (6)	0.0156 (2)	
C25	0.43406 (9)	0.67254 (11)	0.30988 (6)	0.0192 (3)	
H25	0.4223	0.6409	0.2656	0.023*	
C26	0.52380 (9)	0.73073 (11)	0.33227 (6)	0.0206 (3)	
H26	0.5723	0.7386	0.3029	0.025*	
C27	0.54357 (9)	0.77772 (10)	0.39710 (6)	0.0186 (2)	
C28	0.47053 (9)	0.76542 (10)	0.43854 (6)	0.0180 (2)	
H28	0.4822	0.7974	0.4828	0.022*	
C29	0.38064 (9)	0.70720 (10)	0.41650 (6)	0.0167 (2)	
H29	0.3321	0.6994	0.4459	0.020*	
C30	0.64131 (10)	0.84034 (11)	0.42213 (7)	0.0240 (3)	
H30A	0.6909	0.8227	0.3920	0.029*	
H30B	0.6698	0.8133	0.4683	0.029*	
C31	0.62664 (12)	0.96826 (13)	0.42412 (9)	0.0362 (4)	
H31A	0.6050	0.9966	0.3779	0.054*	
H31B	0.6907	1.0043	0.4440	0.054*	
H31C	0.5747	0.9860	0.4517	0.054*	
C32	0.24685 (9)	0.46056 (10)	0.45536 (6)	0.0162 (2)	
C33	0.34447 (9)	0.43509 (10)	0.48903 (6)	0.0186 (2)	
H33	0.3851	0.3832	0.4692	0.022*	
C34	0.38284 (10)	0.48437 (11)	0.55082 (6)	0.0208 (3)	
H34	0.4498	0.4668	0.5723	0.025*	
C35	0.32457 (10)	0.55942 (10)	0.58197 (6)	0.0203 (3)	
C36	0.22587 (10)	0.58115 (11)	0.54989 (6)	0.0213 (3)	
H36	0.1838	0.6294	0.5712	0.026*	
C37	0.18765 (9)	0.53323 (11)	0.48694 (6)	0.0199 (3)	
H37	0.1205	0.5505	0.4655	0.024*	
C38	0.37098 (11)	0.61916 (12)	0.64667 (7)	0.0262 (3)	
H38A	0.4108	0.5643	0.6778	0.031*	
H38B	0.3164	0.6494	0.6694	0.031*	
C39	0.43976 (11)	0.71594 (12)	0.63209 (7)	0.0297 (3)	
H39A	0.4911	0.6869	0.6069	0.045*	
H39B	0.4732	0.7491	0.6750	0.045*	
H39C	0.3991	0.7740	0.6050	0.045*	

### Atomic displacement parameters (Å^2^)

**Table:**

	*U*^11^	*U*^22^	*U*^33^	*U*^12^	*U*^13^	*U*^23^
O1	0.0268 (5)	0.0266 (5)	0.0248 (5)	0.0072 (4)	0.0061 (4)	−0.0061 (4)
N1	0.0160 (5)	0.0227 (5)	0.0141 (5)	−0.0028 (4)	0.0027 (4)	0.0020 (4)
N2	0.0169 (5)	0.0157 (5)	0.0142 (5)	−0.0027 (4)	0.0003 (4)	0.0020 (4)
C1	0.0193 (6)	0.0147 (5)	0.0167 (6)	−0.0024 (4)	0.0015 (5)	−0.0001 (4)
C2	0.0190 (6)	0.0147 (5)	0.0134 (5)	−0.0009 (4)	0.0022 (4)	−0.0015 (4)
C3	0.0160 (5)	0.0151 (5)	0.0134 (5)	−0.0012 (4)	0.0022 (4)	−0.0005 (4)
C4	0.0147 (5)	0.0170 (6)	0.0128 (5)	0.0000 (4)	0.0017 (4)	0.0006 (4)
C5	0.0155 (5)	0.0163 (6)	0.0159 (6)	0.0010 (4)	0.0023 (4)	0.0004 (5)
C6	0.0177 (6)	0.0153 (5)	0.0158 (6)	0.0006 (4)	0.0015 (4)	0.0021 (4)
C7	0.0206 (6)	0.0150 (5)	0.0171 (6)	−0.0006 (4)	0.0065 (5)	0.0019 (5)
C8	0.0186 (6)	0.0174 (6)	0.0164 (6)	−0.0043 (4)	0.0027 (5)	0.0039 (5)
C9	0.0186 (6)	0.0282 (7)	0.0170 (6)	−0.0045 (5)	0.0028 (5)	−0.0023 (5)
C10	0.0190 (6)	0.0302 (7)	0.0217 (6)	−0.0013 (5)	0.0056 (5)	−0.0022 (5)
C11	0.0171 (6)	0.0252 (6)	0.0214 (6)	−0.0064 (5)	0.0021 (5)	0.0044 (5)
C12	0.0251 (6)	0.0199 (6)	0.0200 (6)	−0.0087 (5)	−0.0013 (5)	0.0008 (5)
C13	0.0236 (6)	0.0154 (6)	0.0218 (6)	−0.0047 (5)	0.0024 (5)	−0.0005 (5)
C14	0.0172 (6)	0.0350 (8)	0.0266 (7)	−0.0036 (5)	−0.0001 (5)	0.0022 (6)
C15	0.0277 (7)	0.0431 (9)	0.0424 (9)	0.0038 (7)	0.0022 (6)	0.0170 (7)
C16	0.0210 (6)	0.0128 (5)	0.0142 (6)	−0.0013 (4)	0.0010 (4)	−0.0011 (4)
C17	0.0206 (6)	0.0170 (6)	0.0168 (6)	0.0000 (5)	0.0023 (5)	0.0003 (5)
C18	0.0233 (6)	0.0195 (6)	0.0202 (6)	0.0025 (5)	−0.0019 (5)	−0.0001 (5)
C19	0.0337 (7)	0.0156 (6)	0.0155 (6)	0.0037 (5)	−0.0005 (5)	0.0003 (5)
C20	0.0312 (7)	0.0219 (6)	0.0171 (6)	−0.0003 (5)	0.0075 (5)	0.0017 (5)
C21	0.0228 (6)	0.0203 (6)	0.0183 (6)	−0.0016 (5)	0.0031 (5)	0.0002 (5)
C22	0.0408 (8)	0.0296 (7)	0.0161 (6)	0.0083 (6)	−0.0005 (6)	0.0018 (5)
C23	0.0600 (11)	0.0255 (8)	0.0363 (9)	−0.0081 (7)	−0.0212 (8)	0.0107 (7)
C24	0.0155 (5)	0.0146 (5)	0.0160 (6)	0.0006 (4)	0.0007 (4)	0.0022 (4)
C25	0.0204 (6)	0.0225 (6)	0.0147 (6)	−0.0023 (5)	0.0030 (5)	−0.0002 (5)
C26	0.0180 (6)	0.0249 (6)	0.0196 (6)	−0.0019 (5)	0.0054 (5)	0.0033 (5)
C27	0.0166 (6)	0.0174 (6)	0.0205 (6)	−0.0006 (4)	−0.0007 (5)	0.0032 (5)
C28	0.0201 (6)	0.0173 (6)	0.0152 (6)	0.0001 (5)	−0.0005 (5)	−0.0002 (5)
C29	0.0175 (6)	0.0165 (6)	0.0163 (6)	0.0013 (4)	0.0036 (4)	0.0018 (5)
C30	0.0180 (6)	0.0283 (7)	0.0245 (7)	−0.0052 (5)	0.0003 (5)	0.0010 (5)
C31	0.0313 (8)	0.0279 (8)	0.0468 (9)	−0.0117 (6)	−0.0009 (7)	0.0017 (7)
C32	0.0194 (6)	0.0148 (5)	0.0148 (6)	−0.0012 (4)	0.0038 (4)	0.0039 (4)
C33	0.0217 (6)	0.0166 (6)	0.0177 (6)	0.0035 (5)	0.0039 (5)	0.0021 (5)
C34	0.0221 (6)	0.0207 (6)	0.0184 (6)	0.0022 (5)	0.0004 (5)	0.0036 (5)
C35	0.0277 (6)	0.0191 (6)	0.0143 (6)	−0.0019 (5)	0.0046 (5)	0.0039 (5)
C36	0.0257 (6)	0.0207 (6)	0.0192 (6)	0.0024 (5)	0.0085 (5)	0.0001 (5)
C37	0.0190 (6)	0.0214 (6)	0.0196 (6)	0.0022 (5)	0.0044 (5)	0.0036 (5)
C38	0.0335 (7)	0.0288 (7)	0.0162 (6)	−0.0019 (6)	0.0038 (5)	−0.0013 (5)
C39	0.0353 (8)	0.0311 (7)	0.0237 (7)	−0.0059 (6)	0.0079 (6)	−0.0089 (6)

### Geometric parameters (Å, °)

**Table:**

O1—C7	1.2135 (15)	C19—C20	1.3917 (19)
N1—C6	1.4647 (15)	C19—C22	1.5143 (17)
N1—C1	1.4654 (15)	C20—C21	1.3952 (17)
N1—H1N	0.897 (16)	C20—H20	0.9500
N2—C3	1.4742 (14)	C21—H21	0.9500
N2—C4	1.4763 (15)	C22—C23	1.503 (2)
N2—H2N	0.880 (16)	C22—H22A	0.9900
C1—C8	1.5159 (16)	C22—H22B	0.9900
C1—C2	1.5604 (16)	C23—H23A	0.9800
C1—H1	1.0000	C23—H23B	0.9800
C2—C7	1.5063 (16)	C23—H23C	0.9800
C2—C3	1.5644 (16)	C24—C25	1.3936 (17)
C2—H2	1.0000	C24—C29	1.3957 (17)
C3—C16	1.5158 (16)	C25—C26	1.3909 (17)
C3—H3	1.0000	C25—H25	0.9500
C4—C24	1.5159 (16)	C26—C27	1.3939 (18)
C4—C5	1.5603 (16)	C26—H26	0.9500
C4—H4	1.0000	C27—C28	1.3910 (18)
C5—C7	1.5073 (16)	C27—C30	1.5130 (17)
C5—C6	1.5591 (16)	C28—C29	1.3916 (17)
C5—H5	1.0000	C28—H28	0.9500
C6—C32	1.5172 (16)	C29—H29	0.9500
C6—H6	1.0000	C30—C31	1.526 (2)
C8—C9	1.3901 (18)	C30—H30A	0.9900
C8—C13	1.3950 (17)	C30—H30B	0.9900
C9—C10	1.3985 (17)	C31—H31A	0.9800
C9—H9	0.9500	C31—H31B	0.9800
C10—C11	1.3911 (18)	C31—H31C	0.9800
C10—H10	0.9500	C32—C37	1.3900 (17)
C11—C12	1.3940 (19)	C32—C33	1.3981 (16)
C11—C14	1.5123 (17)	C33—C34	1.3840 (17)
C12—C13	1.3880 (18)	C33—H33	0.9500
C12—H12	0.9500	C34—C35	1.3949 (18)
C13—H13	0.9500	C34—H34	0.9500
C14—C15	1.519 (2)	C35—C36	1.3917 (18)
C14—H14A	0.9900	C35—C38	1.5123 (18)
C14—H14B	0.9900	C36—C37	1.3962 (18)
C15—H15A	0.9800	C36—H36	0.9500
C15—H15B	0.9800	C37—H37	0.9500
C15—H15C	0.9800	C38—C39	1.5269 (19)
C16—C21	1.3889 (17)	C38—H38A	0.9900
C16—C17	1.3964 (16)	C38—H38B	0.9900
C17—C18	1.3909 (17)	C39—H39A	0.9800
C17—H17	0.9500	C39—H39B	0.9800
C18—C19	1.3899 (19)	C39—H39C	0.9800
C18—H18	0.9500		
			
C6—N1—C1	114.38 (10)	C17—C18—H18	119.5
C6—N1—H1N	109.3 (10)	C18—C19—C20	117.86 (11)
C1—N1—H1N	108.3 (10)	C18—C19—C22	120.48 (12)
C3—N2—C4	111.13 (9)	C20—C19—C22	121.65 (12)
C3—N2—H2N	106.6 (10)	C19—C20—C21	121.40 (12)
C4—N2—H2N	109.2 (10)	C19—C20—H20	119.3
N1—C1—C8	111.55 (10)	C21—C20—H20	119.3
N1—C1—C2	109.23 (9)	C16—C21—C20	120.44 (12)
C8—C1—C2	109.68 (9)	C16—C21—H21	119.8
N1—C1—H1	108.8	C20—C21—H21	119.8
C8—C1—H1	108.8	C23—C22—C19	113.60 (12)
C2—C1—H1	108.8	C23—C22—H22A	108.8
C7—C2—C1	105.29 (9)	C19—C22—H22A	108.8
C7—C2—C3	109.69 (9)	C23—C22—H22B	108.8
C1—C2—C3	113.73 (10)	C19—C22—H22B	108.8
C7—C2—H2	109.3	H22A—C22—H22B	107.7
C1—C2—H2	109.3	C22—C23—H23A	109.5
C3—C2—H2	109.3	C22—C23—H23B	109.5
N2—C3—C16	111.00 (9)	H23A—C23—H23B	109.5
N2—C3—C2	107.31 (9)	C22—C23—H23C	109.5
C16—C3—C2	110.51 (9)	H23A—C23—H23C	109.5
N2—C3—H3	109.3	H23B—C23—H23C	109.5
C16—C3—H3	109.3	C25—C24—C29	118.27 (11)
C2—C3—H3	109.3	C25—C24—C4	121.40 (10)
N2—C4—C24	110.15 (9)	C29—C24—C4	120.33 (10)
N2—C4—C5	107.32 (9)	C26—C25—C24	120.87 (11)
C24—C4—C5	112.23 (9)	C26—C25—H25	119.6
N2—C4—H4	109.0	C24—C25—H25	119.6
C24—C4—H4	109.0	C25—C26—C27	121.00 (11)
C5—C4—H4	109.0	C25—C26—H26	119.5
C7—C5—C6	105.52 (9)	C27—C26—H26	119.5
C7—C5—C4	109.73 (9)	C28—C27—C26	118.00 (11)
C6—C5—C4	112.51 (10)	C28—C27—C30	120.60 (11)
C7—C5—H5	109.7	C26—C27—C30	121.40 (11)
C6—C5—H5	109.7	C27—C28—C29	121.30 (11)
C4—C5—H5	109.7	C27—C28—H28	119.3
N1—C6—C32	111.99 (10)	C29—C28—H28	119.3
N1—C6—C5	108.68 (9)	C28—C29—C24	120.55 (11)
C32—C6—C5	109.75 (9)	C28—C29—H29	119.7
N1—C6—H6	108.8	C24—C29—H29	119.7
C32—C6—H6	108.8	C27—C30—C31	112.68 (11)
C5—C6—H6	108.8	C27—C30—H30A	109.1
O1—C7—C2	124.19 (11)	C31—C30—H30A	109.1
O1—C7—C5	124.30 (11)	C27—C30—H30B	109.1
C2—C7—C5	111.01 (10)	C31—C30—H30B	109.1
C9—C8—C13	118.28 (11)	H30A—C30—H30B	107.8
C9—C8—C1	122.57 (11)	C30—C31—H31A	109.5
C13—C8—C1	118.94 (11)	C30—C31—H31B	109.5
C8—C9—C10	120.74 (12)	H31A—C31—H31B	109.5
C8—C9—H9	119.6	C30—C31—H31C	109.5
C10—C9—H9	119.6	H31A—C31—H31C	109.5
C11—C10—C9	120.91 (12)	H31B—C31—H31C	109.5
C11—C10—H10	119.5	C37—C32—C33	118.18 (11)
C9—C10—H10	119.5	C37—C32—C6	123.36 (11)
C10—C11—C12	118.07 (12)	C33—C32—C6	118.38 (11)
C10—C11—C14	121.40 (12)	C34—C33—C32	121.03 (11)
C12—C11—C14	120.51 (12)	C34—C33—H33	119.5
C13—C12—C11	121.12 (12)	C32—C33—H33	119.5
C13—C12—H12	119.4	C33—C34—C35	120.98 (12)
C11—C12—H12	119.4	C33—C34—H34	119.5
C12—C13—C8	120.84 (12)	C35—C34—H34	119.5
C12—C13—H13	119.6	C36—C35—C34	118.04 (11)
C8—C13—H13	119.6	C36—C35—C38	121.96 (12)
C11—C14—C15	111.77 (11)	C34—C35—C38	119.91 (12)
C11—C14—H14A	109.3	C35—C36—C37	121.06 (12)
C15—C14—H14A	109.3	C35—C36—H36	119.5
C11—C14—H14B	109.3	C37—C36—H36	119.5
C15—C14—H14B	109.3	C32—C37—C36	120.64 (11)
H14A—C14—H14B	107.9	C32—C37—H37	119.7
C14—C15—H15A	109.5	C36—C37—H37	119.7
C14—C15—H15B	109.5	C35—C38—C39	111.14 (11)
H15A—C15—H15B	109.5	C35—C38—H38A	109.4
C14—C15—H15C	109.5	C39—C38—H38A	109.4
H15A—C15—H15C	109.5	C35—C38—H38B	109.4
H15B—C15—H15C	109.5	C39—C38—H38B	109.4
C21—C16—C17	118.34 (11)	H38A—C38—H38B	108.0
C21—C16—C3	121.71 (11)	C38—C39—H39A	109.5
C17—C16—C3	119.74 (10)	C38—C39—H39B	109.5
C18—C17—C16	120.82 (12)	H39A—C39—H39B	109.5
C18—C17—H17	119.6	C38—C39—H39C	109.5
C16—C17—H17	119.6	H39A—C39—H39C	109.5
C19—C18—C17	121.08 (12)	H39B—C39—H39C	109.5
C19—C18—H18	119.5		
			
C6—N1—C1—C8	179.96 (9)	N2—C3—C16—C21	31.23 (15)
C6—N1—C1—C2	−58.63 (13)	C2—C3—C16—C21	−87.71 (13)
N1—C1—C2—C7	58.31 (12)	N2—C3—C16—C17	−154.06 (11)
C8—C1—C2—C7	−179.15 (9)	C2—C3—C16—C17	86.99 (13)
N1—C1—C2—C3	−61.80 (12)	C21—C16—C17—C18	1.90 (18)
C8—C1—C2—C3	60.74 (12)	C3—C16—C17—C18	−172.98 (11)
C4—N2—C3—C16	175.29 (9)	C16—C17—C18—C19	0.23 (19)
C4—N2—C3—C2	−63.86 (12)	C17—C18—C19—C20	−2.14 (19)
C7—C2—C3—N2	1.01 (12)	C17—C18—C19—C22	176.72 (12)
C1—C2—C3—N2	118.60 (10)	C18—C19—C20—C21	1.95 (19)
C7—C2—C3—C16	122.17 (10)	C22—C19—C20—C21	−176.90 (12)
C1—C2—C3—C16	−120.24 (11)	C17—C16—C21—C20	−2.09 (18)
C3—N2—C4—C24	−171.74 (9)	C3—C16—C21—C20	172.69 (11)
C3—N2—C4—C5	65.81 (12)	C19—C20—C21—C16	0.17 (19)
N2—C4—C5—C7	−4.20 (12)	C18—C19—C22—C23	81.21 (18)
C24—C4—C5—C7	−125.34 (10)	C20—C19—C22—C23	−99.97 (16)
N2—C4—C5—C6	−121.35 (10)	N2—C4—C24—C25	−38.85 (15)
C24—C4—C5—C6	117.50 (11)	C5—C4—C24—C25	80.66 (14)
C1—N1—C6—C32	−179.85 (9)	N2—C4—C24—C29	141.59 (11)
C1—N1—C6—C5	58.74 (13)	C5—C4—C24—C29	−98.90 (13)
C7—C5—C6—N1	−58.93 (12)	C29—C24—C25—C26	−0.23 (18)
C4—C5—C6—N1	60.69 (12)	C4—C24—C25—C26	−179.80 (11)
C7—C5—C6—C32	178.30 (9)	C24—C25—C26—C27	0.33 (19)
C4—C5—C6—C32	−62.08 (12)	C25—C26—C27—C28	−0.48 (18)
C1—C2—C7—O1	106.88 (13)	C25—C26—C27—C30	179.50 (12)
C3—C2—C7—O1	−130.38 (12)	C26—C27—C28—C29	0.56 (18)
C1—C2—C7—C5	−65.30 (12)	C30—C27—C28—C29	−179.42 (11)
C3—C2—C7—C5	57.44 (12)	C27—C28—C29—C24	−0.49 (18)
C6—C5—C7—O1	−106.30 (13)	C25—C24—C29—C28	0.31 (17)
C4—C5—C7—O1	132.25 (12)	C4—C24—C29—C28	179.88 (11)
C6—C5—C7—C2	65.88 (12)	C28—C27—C30—C31	−74.32 (16)
C4—C5—C7—C2	−55.57 (12)	C26—C27—C30—C31	105.70 (15)
N1—C1—C8—C9	16.87 (16)	N1—C6—C32—C37	−10.32 (16)
C2—C1—C8—C9	−104.28 (13)	C5—C6—C32—C37	110.47 (13)
N1—C1—C8—C13	−168.51 (10)	N1—C6—C32—C33	173.07 (10)
C2—C1—C8—C13	70.33 (14)	C5—C6—C32—C33	−66.13 (13)
C13—C8—C9—C10	−1.58 (18)	C37—C32—C33—C34	−2.61 (18)
C1—C8—C9—C10	173.07 (11)	C6—C32—C33—C34	174.17 (11)
C8—C9—C10—C11	−0.2 (2)	C32—C33—C34—C35	1.16 (19)
C9—C10—C11—C12	1.33 (19)	C33—C34—C35—C36	1.54 (18)
C9—C10—C11—C14	−176.85 (12)	C33—C34—C35—C38	−175.19 (12)
C10—C11—C12—C13	−0.73 (19)	C34—C35—C36—C37	−2.78 (19)
C14—C11—C12—C13	177.46 (12)	C38—C35—C36—C37	173.88 (12)
C11—C12—C13—C8	−1.04 (19)	C33—C32—C37—C36	1.37 (18)
C9—C8—C13—C12	2.18 (18)	C6—C32—C37—C36	−175.24 (11)
C1—C8—C13—C12	−172.67 (11)	C35—C36—C37—C32	1.33 (19)
C10—C11—C14—C15	105.45 (15)	C36—C35—C38—C39	−100.09 (15)
C12—C11—C14—C15	−72.69 (16)	C34—C35—C38—C39	76.51 (15)

### Hydrogen-bond geometry (Å, °)

**Table:**

*D*—H···*A*	*D*—H	H···*A*	*D*···*A*	*D*—H···*A*
C26—H26···O1^i^	0.95	2.51	3.3837 (16)	153

Symmetry codes: (i) −*x*+1, *y*+1/2, −*z*+1/2.
